# Editorial: A moving target: exploring if, when, how, and why promoting quality of life counts among children and adolescents during COVID-19 pandemic

**DOI:** 10.3389/fpubh.2023.1339945

**Published:** 2023-12-14

**Authors:** Yuan Hao, Xixi Sun, Wenjie Duan, Daniel Y. T. Fong, Xuejing Jin

**Affiliations:** ^1^School of Social and Public Administration, East China University of Science and Technology, Shanghai, China; ^2^School of Nursing, The University of Hong Kong, Hong Kong, China; ^3^Centre for Evidence-Based Chinese Medicine, Beijing University of Chinese Medicine, Beijing, China

**Keywords:** quality of life, children, adolescents, promotion, assessment

## Introduction

Quality of life (QoL) is an important outcome in health and medical promotion (1, 2). Many scholars are interested in QoL, targeting various patient groups (2), such as chronic illnesses (3), spinal cord injury, and alcohol abuse and dependence (1, 4–6).

QoL research on children and adolescents has been focusing on mental health problems (7, 8), overweight and obesity (9), health-related QoL (HRQoL) (10), self-harm and suicidal behaviors (11), etc. Such studies usually pay more attention on traditional health indicators. However, there is a need to explore the positive elements that promote QoL of children and adolescents. Therefore, the “moving target” perspective is adopted.

The COVID-19 pandemic has brought about significant psychological, physical fitness, and lifestyle challenges for children and adolescents (12), including insomnia (13), anxiety (14), social exclusion (15, 16), post-traumatic stress (17), reduced social activities, and prolonged sitting and screen time (18). These challenges had diverse impacts on the QoL of children and adolescents.

## “If” and “how” to promote QoL of children and adolescents

Addressing the questions of “if” and “how” infection control measures affect the romantic relationships, mental health, post-infection trauma status, and use of social network in children and adolescents.

Fujino et al. designed a prospective cohort to study the change in single youth's romantic relationships during COVID-19. Research found that stricter infection control in the workplace was correlated with an increased initiation of romantic relationships.

A longitudinal study conducted by Zhu et al. studied whether and how relocated adolescents were affected by changes in their living environment during the COVID-19 pandemic. It revealed that the psychological resilience and mental health problems of relocated adolescents are a two-way, mutually influential dynamic.

Xu et al. designed a latent profile study to explore the impact of family functioning on post-traumatic reactions in infected adolescents during COVID-19. This study identified three classes: the growth class, the struggling class, and the pain class. The growth and struggle classes were influenced by problem solving and behavioral control in family functioning, while the growth and pain classes were influenced by problem solving, role, behavioral control, and total functioning. The pain and struggle classes were also influenced by problem-solving and roles.

Tremolada et al. investigated the type of social network use and the effects of the COVID-19 pandemic. It was shown that adolescents in the clinical groups show passive use of social networks, while healthy adolescents use them more actively. It also found that those with high levels of social anxiety are more likely to be negatively affected by COVID-19.

These articles show that the COVID-19 pandemic has influenced the QoL of children and adolescents, primarily on social relationships, mental health, and trauma, both positively and negatively. On one hand, it restricts the social interactions of children and adolescents, and increases psychological problems and trauma. On the other hand, it encourages single youth to be more willing to pursue intimate relationships, and promotes adolescent psychological resilience and traumatized growth.

## “When” and “why” to promote children and adolescents

Three articles explored the timing and reasons behind promoting the QoL of children and adolescents.

Lee et al. analyzed data collected during 2017–2021 to examine trends in health behaviors and mental health among adolescents. It reported a reduction in alcohol consumption and insufficient physical activity among adolescents during the first 2 years of the COVID-19 pandemic. However, obesity rates among adolescents had risen. Furthermore, the incidence of mental health problems among all adolescents decreased in 2020 but rose again in 2021. In adolescents from low-income families, the incidence of mental health problems was the highest over 5 years since the COVID-19 outbreak.

A prospective cohort study was conducted by Paton et al. to investigate the mental health trajectories of college students during COVID-19. Nearly two-thirds of the students who were “constants” and “thrivers” maintained a stable trend in mental health. The two groups of students, “strugglers” and “decliners”, accounted for about 30% and generally showed at least a slight decline in their well-being. The students who were “improvers” showed a significant improvement in wellbeing.

A comparative study was conducted by Raffagnato et al. to study the impact of adolescent mental health problems on their hospital admissions and readmissions. It showed a gradual increased use of substances and electronic devices, resulting in a significant increase in hospital readmissions.

These works indicate that adolescents' health behaviors and mental health change over time, and different groups may exhibit different trends. The identified risk factors of adolescents' QoL are important for developing QoL interventions for adolescents.

## Future research

QoL is an important indicator of youth development (19). The collection of these Research Topics focuses on the QoL of children and adolescents across different age groups and fields (19). These analyses provide important information for understanding the current statuses, trends, and implications for the development of future interventions of COVID-19 to enhance adolescents' QoL.

QoL of children and adolescents is influenced by their developmental stage and specific life events. The key considerations are the early identification of risk factors and operation of effective interventions (14), and focus on both short- and long-term benefits to children and adolescents (20). However, it remains unclear of what are the effective identification processes and interventions. Furthermore, the mechanism underlying the influences of COVID-19 on QoL in children and adolescents is also unclear. Finally, the critical issue of how to provide adolescents with social resources and services to promote their QoL has not been adequately addressed either. Thus, from the “a moving target” perspective, there is still a need for developing and evaluating more high-quality interventions to promote QoL in different contexts.

## Author contributions

YH: Writing—original draft. XS: Writing—original draft. WD: Writing—review & editing, Conceptualization, Funding acquisition, Project administration, Supervision. DF: Writing—review & editing. XJ: Writing—review & editing.
